# High‐Caloric Realimentation and Mental and Physical Well‐Being in Patients With Extreme Anorexia Nervosa. A Prospective Study

**DOI:** 10.1002/erv.70074

**Published:** 2025-12-25

**Authors:** Ulrich Voderholzer, Juliane Silbernagl, Verena Haas, Ulrich Cuntz, Silke Naab, Norbert Quadflieg

**Affiliations:** ^1^ Department of Psychiatry and Psychotherapy University Hospital Ludwig‐Maximilians‐University (LMU) Munich Germany; ^2^ Schoen Clinic Roseneck Prien Germany; ^3^ Department of Psychiatry and Psychotherapy University Hospital of Freiburg Freiburg im Breisgau Germany; ^4^ Department of Child and Adolescent Psychiatry Charité‐Universitätsmedizin Berlin Berlin Germany; ^5^ Paracelsus Medical University Salzburg Austria

**Keywords:** anorexia nervosa, eating disorders, high‐caloric realimentation, outcome, psychological symptoms

## Abstract

**Objective:**

While high caloric realimentation (HCR) is increasingly recommended for quick weight restoration of patients with anorexia nervosa (AN), the development of AN‐specific psychopathology and somatic symptoms during HCR have so far insufficiently been studied.

**Method:**

Patients hospitalised for the treatment of AN received oral realimentation starting with 2000 kcal under close medical monitoring and a substitution regimen. Weekly over the initial 6 weeks of treatment, assessments included body weight, drive for thinnesss, body dissatisfaction, depression, and somatic symptoms.

**Results:**

In 46 patients (mean age 27.57 years, 2 males, 44 females), the body mass index increased from 12.6 (SD 1.1) at baseline to 14.4 (SD 1.0) kg/m^2^ (*p* < 0.001) at week 6 (*d* = 2.57). Drive for thinnesss did not change (28.9 [SD 10.2] to 27.7 [SD 10.8]; *p* = 0.102), while body dissatisfaction increased slightly from 37.3 (SD 7.8) to 39.1 (SD 9.2; *p* = 0.019; *d* = 0.41). Depression and somatic symptoms decreased significantly (*p* < 0.001) with large effect sizes (*d* = 1.09, *d* = 0.90, respectively). No case of refeeding syndrome was encountered.

**Discussion:**

HCR was medically safe and associated with a decrease in depressive and somatic symptoms at only slight increase in body dissatisfaction. Fast realimentation brings patients with extreme AN quickly out of a weight range dangerous to their health.

## Key Points

- Bringing the patient in a more healthy weight range is a primary aim of treatment for anorexia nervosa
- Quick high‐caloric realimentation with prophylactic phosphate and thiamine substitution under close clinical surveillance is safe
- Quick weight gain under clinical surveillance does not negatively affect mental and physical well‐being of patients
- Depression, gastrointestinal afflictions and somatic complaints decreased during treatment and quick realimentantion

## 1 Introduction

One of the key clinical symptoms of anorexia nervosa (AN) is significantly low body weight. Very low body weight affects multiple organs with a high prevalence of serious medical problems (Gaudiani et al. 2012) including metabolic, cardiopulmonary, endocrine, gastrointestinal and haematological complications (Cass et al. 2020). Consequently, weight restoration to a healthy level is a major focus in the treatment of AN, and is mentioned as a central aim of treatment in international guidelines (S3, NICE, APA). Considering that discharge from hospital treatment with a low body weight is associated with a higher re‐hospitalisation rate (Baran et al. 1995; Frostad et al. 2022), this indicates a requirement to initiate quick restoration to a higher body weight to bring the patients sooner into a healthier weight range, helping to avert grave negative consequences of very low weight. International guidelines differ on the recommended weekly weight gain, ranging from 0.5 to 2.0 kg/week (Herpertz‐Dahlmann et al. 2015). More recent research, however, suggests that high‐caloric realimentation (HCR) in the initial phase of treatment is possible and medically safe (Dalenbrook et al. 2022; Gaudiani et al. 2016; Koerner et al. 2020; Pettersson et al. 2016; Smith et al. 2016), provided that close clinical surveillance and phosphate substitution are ensured (Haas et al. 2021; Garber et al. 2021; Madden, Miskovic‐Wheatley, Clarke, et al. 2015a). Systematic reviews confirmed these findings (Garber et al. 2016; Mosuka et al. 2023). In addition, Gaudiani et al. (2016) showed that early realimentation for severe AN is equally possible in all age groups from late adolescence to patients over 40 years old.

While studies generally reported the weight gain during and after HCR (Cuntz et al. 2022; Dalenbrook et al. 2022; Garber et al. 2012, 2021; Gjoertz et al., 2020; Hemmingsen et al. 2022; Kezelman et al. 2018; Koerner et al. 2020; Madden, Miskovic‐Wheatley, Clarke, et al. 2015a; Mattar et al. 2012; Pettersson et al. 2016), few studies addressed the association of realimentation and psychological symptoms (Accurso et al. 2023; Akgül et al. 2022; Hemmingsen et al. 2022; Kezelman et al. 2018; Salter et al. 2024). Fear of gaining weight and body image distortion are central clinical features of AN. Thus, a rapid increase of body weight could be expected to be associated with an intensifying drive for thinnesss and dissatisfaction with the increasingly heavier body. Similarly, depression and somatic symptoms (including gastro‐intestinal symptoms) would also be expected to increase, reflecting the patients' efforts to manage the weight increase of their own body. On the other hand, data from several studies showed a decrease of ED‐specific symptoms, depression, somatisation, somatic and gastro‐intestinal symptoms being associated with an increase of body weight over the course of intensive inpatient treatment including psychotherapy (Riedlinger et al. 2022; Quadflieg et al. 2023; Schlegl et al. 2014). To address this gap in the literature, participants of a HCR programme were assessed over the first 6 weeks of treatment by weekly self‐report questionnaires covering both ED‐specific and general psychopathology. The HCR was continued for a longer time, but we felt the initial 6‐week period to be adequate for addressing our research question. The aim of the study was to assess changes in psychological and somatic symptoms during HCR.

## 2 Method

### 2.1 Participants

All participants were treated at an ED unit of the Schoen Clinic Roseneck in Prien am Chiemsee, Germany, spezialized in severe and complex AN cases. Inclusion criteria were (1) a diagnosis of AN or atypical AN; (2) BMI ≤ 14 kg/m^2^ or for minors a BMI below the third percentile for age; (3) age at admission at least 12 years; (4) written informed consent by all participants and legal carers of minors. Pregnant patients and patients with acute psychosis were excluded from the realimentation programme. Severe cognitive limitations preventing a useful assessment by questionnaire were an additional exclusion criterion.

### 2.2 Realimentation

For severely underweight patients with AN the Schoen Clinic Roseneck offers—with the consent of the patient and their legal carers where appropriate—a rapid HCR programme to achieve quick weight restoration or at least a quick weight increase. The aim was to enable the patient to subsequently participate in the regular treatment programme of the clinic. Intensive treatment aimed at a weight gain of 0.7–1.0 kg per week (or 0.1 kg per day) by a regimen of consuming at least 2000 kcal from the first day of treatment. No differences were made between adolescents and adults. Patients were weighted daily. Over the course of the therapy, most patients needed a significantly higher caloric input to achieve the projected weight gain, and for these patients the amount of calories was increased up to more than 4000 kcal. No standardised protocol for caloric intake increase was applied. If the projected weight increase was not achieved, the portion size of the main meal was increased individually, or additional liquid drinks or snack meals were prescribed. In extreme cases, nasogastric or percutaneous endoscopic gastrostomy tube feeding with the consent of the patient or legal guardian was applied.

The nutritional pattern provided for three main meals per day, with up to three additional snacks when necessary. Meals consisted of approximately 39% carbohydrates, 42% fats and 19% proteins. If necessary, caloric intake was supplemented by up to three flasks per day of high‐caloric fluids (e.g., Fresubin 200 mL per flask with 2 kcal/mL; Cuntz et al. 2022) in order to ensure an adequate caloric intake. Meals were taken in fixed groups of patients and the adherence to the nutritional regimen was observed by the clinical staff. Weekly group sessions evaluating the clinical progress and restrictions in exercise supported these measures. The ward staff prevented purging behaviour and excessive exercise as far as possible. This was achieved through video surveillance of the rooms and, if necessary, restricted access to the toilets. The HCR programme lasted longer than the first 6 weeks reported here, and patients remained in this ward until their weight had stabilised sufficiently (usually a target BMI of 14 kg/m^2^) and they were able to control purging behaviour and excessive exercise themselves. The patients were then transferred to the clinic's normal inpatient ED programme or to other facilities that specialise in EDs. A detailed description of the HCR programme is provided by Dalenbrook et al. (2022).

Weekly or more often monitoring of electrolytes (sodium, potassium, phosphate, magnesium, chloride), creatine kinase, transaminases, gamma‐glutamyl‐transferase (‐GT), creatinine, urea, as well as blood count (without white blood cell differential) was provided for all patients. In addition, electrocardiogram, haematocrit, blood pressure, heart rate and oedema status were monitored closely. Thiamine (200 mg/d) and phosphate (612–1224 mg/d) were supplemented routinely for two weeks and phosphate thereafter if needed. The administration of diuretics was necessary at times in some patients, when either the heart rate per minute exceeded the systolic blood pressure in mmHg or the severity of the oedema was subjectively too stressful. Low serum electrolyte concentrations of phosphate (< 0.32 mmol/L), magnesium (< 0.50 mmol/L) and potassium (< 2.5 mmol/L) were considered as indicating impending refeeding syndrome for all age groups. More details can be found in Dalenbrook et al. (2022).

### 2.3 Assessments

Participants were assessed at the beginning of treatment (T0) and once each week over the first 6 weeks of treatment (T1–T6). The time frame was the last 7 days before assessment and included the following questionnaires:

Two subscales of the Patient Health Questionnaire (PHQ; Spitzer et al. 1999; Löwe et al. 2002). Subscale Depression (9 items; Cronbach's *α* in present sample = 0.889) covers symptoms like decreased interest in activities or negative self‐evaluation) and subscale Somatic symptoms (14 items; *α* = 0.862; one item asking for pain or problems in sexual intercourse was omitted as not relevant during inpatient treatment) covers body‐related complaints like back pain, feeling sick, feeling tyred, etc.

Eating Disorder Inventory (EDI; Garner et al. 1983; Paul and Thiel 2005) subscales drive for thinnesss (7 Items, *α* = 0.932) and body dissatisfaction (9 Items, *α* = 0.780).

Gastro‐Questionnaire (Leibbrand et al. 2002) severity score of gastro‐intestinal complaints based on frequency of symptoms and intensity of subjective suffering; (24 items, range 0–96; *α* = 0.889).

Adherence to the treatment regimen was assessed by the staff on a scale including the ratings 0 = very good (patient adheres to all prescriptions and rules), 1 = slight deviations from prescriptions and rules, 2 = moderate (at times deviations from prescriptions and rules), 3 = poor (frequent deviations from prescriptions and rules), 4 = very poor (significant deviations from prescriptions and rules), and 5 = none (patients does not adhere to prescriptions and rules). Drive for exercise was assessed by the staff on a scale ranging from 0 (none) to 4 (very strong).

### 2.4 Statistical Analyses

Means with standard deviations are reported for the seven time points of measurement and analyses of variance tested for overall time effects. Post‐hoc paired *t*‐tests compared T1‐T6 values with the T0 baseline value. Cohen's *d* was computed for pairwise comparisons, with *d* = 0.2 indicating small effects, *d* = 0.5 medium effects, and *d* = 0.8 large effects (Cohen 1988). Due to the small number of drop‐outs, completer analyses (*N* = 46 or *N* = 40 [PHQ]) are reported. Only four of the 46 completers were below age 18, precluding separate analyses for adolescents and adults. In clinical practice, the BMI is not used for adolescents. To account for this, BMI is reported for adults (*N* = 42) only. In addition, z‐scores based on norms for the German population (Kromeyer‐Hauschild et al. 2001) are reported for the completer sample. Considering the results from large inpatient treatment studies we hypothesised that the rapid weight gain would be associated with improved psychological symptoms as well as improved somatic status.

To identify correlates of BMI change (T6–T0) over the realimentation period we computed correlation coefficients for a large number of admission variables: Age, EDI and PHQ scores, gastro severity score and drive for exercise as described above. In addition scores from the Beck‐Depression‐Inventory‐II (Hautzinger et al. 2009) Brief Symptom Inventory (Franke 2000), Commitment to Exercise Scale (Zeeck et al. 2017), Compulsive Exercise Test (Taranis et al. 2011), Brief Resilience Scale (Chmitorz et al. 2018), Satisfaction With Life Scale (Diener et al. 1985) were taken from the hospital admission documentation. We also included the BMI change in the first 2 weeks of treatment as an indicator of rapid response to realimentation. The same correlates were used for computing correlation coefficients with change scores (T6–T0) of drive for thinness and body dissatisfaction.

## 3 Results

A total of 61 patients were screened positively for study participation. After further information on the study, 10 patients did not consent to participate, and 51 patients (49 female, 2 male according to sex assigned at birth) were included in the study after providing written informed consent. Thirty‐two patients (62.7%) were treated for AN restricting type, 13 patients (25.5%) for AN binge‐eating/purging type, and six patients (11.8%) for atypical AN. One patient terminated study participation after week 1 because she was unwilling to bear the additional effort associated with the study. Due to a lack of motivation for treatment, two patients left the clinic after week 1, and one patient each left the clinic after week 2 and week 3. Thus, the study sample comprised 46 participants (44 females, 2 males). The study was reviewed and approved by the ethics committee of the University of Munich (no. 21‐0486; August 27, 2021).

**FIGURE 1:**
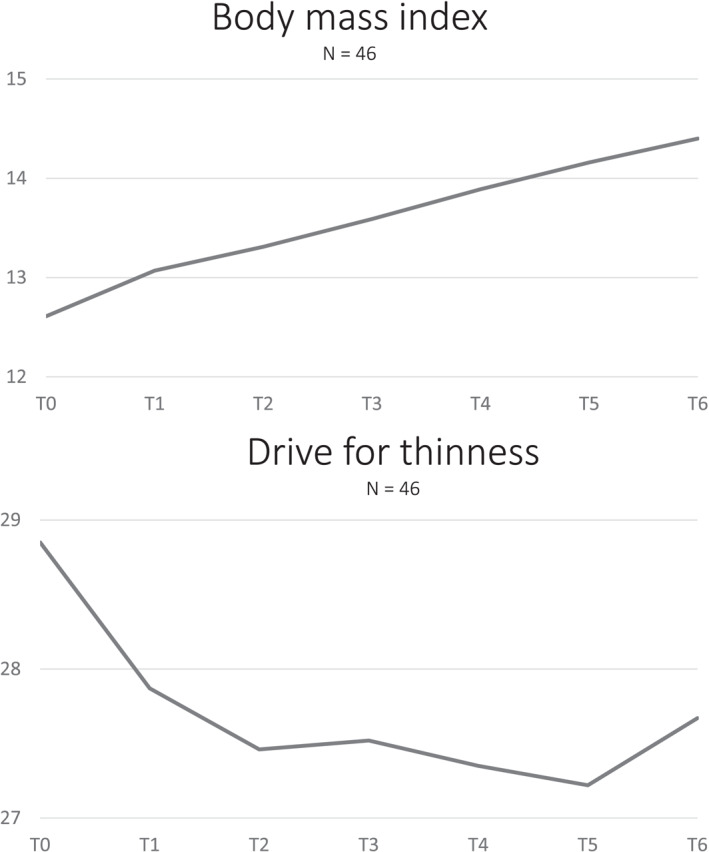
Course of eating disorder symptoms during high‐caloric realimentation in anorexia nervosa (T0 = baseline, T1–T6 = week 1 to week 6 of treatment, respectively).

Mean age of the beginning of treatment was 27.57 (SD 10.89; range 13–62 with four patients below age 18) years. No cases of refeeding syndrome occurred during the first 6 weeks of realimentation. The BMI at admission ranged between 9.2 and 14.0 with a mean value of 12.61 (SD 1.05) kg/m^2^. For more details on the baseline assessments of the sample, see Table S1. Six patients (11.8%) reported purging behaviour at the beginning of treatment, this percentage decreased to 2.2% at the end of the study. Table 1 gives an overview of the course of body weight, drive for exercise, drive for thinnesss and body dissatisfaction. During HCR the BMI of adults increased significantly from 12.56 (SD 1.03) kg/m^2^ at baseline to 14.37 (SD 1.00) kg/m^2^ after the first 6 weeks of HCR. For the total group including adolescents z‐scores increased correspondingly. Compared to baseline, the BMI and the z‐scores increased significantly each week with high effect sizes. Body weight increased from 34.65 (SD 4.24) kg to 39.57 (SD 4.35) kg with an overall weight gain from 0.30 to 10.40 kg (mean weight gain 4.92 [SD 1.95] kg). At the same time, body dissatisfaction increased, but effect sizes were small to medium (Table 1 and Figure 1). From week four, scores were significantly higher than at baseline, indicating that rising body dissatisfaction is delayed relative to increased body weight. Drive for thinnesss scores and drive for exercise scores showed no significant changes over time.

**TABLE 1:** Course of eating disorder symptoms over high‐caloric refeeding in anorexia nervosa (*N* = 46).

	T0 Mean (SD)	T1 Mean (SD)	T2 Mean (SD)	T3 Mean (SD)	T4 Mean (SD)	T5 Mean (SD)	T6 Mean (SD)	ANOVA F (df = 6; 270)	Comparisons versus T0			
										*t* (df = 45)	*p*	*d*
Body Mass index (*N* = 42)^a^	12.56 (1.03)	13.03 (1.14)	13.28 (1.05)	13.55 (1.02)	13.85 (0.99)	14.12 (1.01)	14.37 (1.00)	131.09 *p* < 0.001	T1 versus T0	4.34	< 0.001	0.67
									T2 versus T0	6.98	< 0.001	1.08
									T3 versus T0	9.39	< 0.001	1.45
									T4 versus T0	12.83	< 0.001	1.98
									T5 versus T0	15.02	< 0.001	2.32
									T6 versus T0	17.01	< 0.001	2.62
Body weight z‐scores	−5.85 (1.47)	−5.34 (1.39)	−5.07 (1.26)	−4.78 (1.16)	−4.48 (1.09)	−4.23 (1.09)	−4.01 (1.04)	122.00 *p* < 0.001	T1 versus T0	4.54	< 0.001	0.67
									T2 versus T0	6.94	< 0.001	1.02
									T3 versus T0	9.08	< 0.001	1.34
									T4 versus T0	11.63	< 0.001	1.72
									T5 versus T0	13.66	< 0.001	2.01
									T6 versus T0	14.81	< 0.001	2.18
Drive for exercise	3.11 (2.12)	3.15 (2.08)	3.11 (2.13)	3.26 (2.06)	3.17 (2.06)	3.09 (2.04)	3.11 (2.10)	0.26 *p* = 0.953	T1 versus T0	0.81	0.420	0.12
									T2 versus T0	0.00	1.00	0.00
									T3 versus T0	1.23	0.227	0.18
									T4 versus T0	0.35	0.726	0.05
									T5 versus T0	0.12	0.904	0.02
									T6 versus T0	0.00	1.00	0.00
Drive for thinness	28.85 (10.21)	27.87 (10.34)	27.46 (10.42)	27.52 (10.67)	27.35 (10.09)	27.22 (10.68)	27.67 (10.80)	1.80 *p* = 0.102	T1 versus T0	2.29	0.027	0.34
									T2 versus T0	2.76	0.008	0.41
									T3 versus T0	2.16	0.037	0.32
									T4 versus T0	2.26	0.029	0.33
									T5 versus T0	2.54	0.015	0.38
									T6 versus T0	1.59	0.120	0.23
Body dissatisfaction	37.33 (7.75)	37.91 (7.68)	38.17 (8.62)	38.43 (9.28)	38.70 (8.56)	38.91 (8.64)	39.09 (9.21)	2.60 *p* = 0.019	T1 versus T0	1.12	0.270	0.17
									T2 versus T0	1.72	0.092	0.25
									T3 versus T0	1.82	0.076	0.27
									T4 versus T0	2.76	0.009	0.41
									T5 versus T0	2.68	0.010	0.40
									T6 versus T0	2.75	0.009	0.41

*Note:* T0 = Baseline at beginning of treatment; T1–T6 = week 1‐week 6 of treatment (respectively).

Abbreviation: SD = standard deviation.

^a^ Excluding four patients aged below 18 years; ANOVA df = 6246; *t*‐tests df = 41.

**FIGURE 2:**
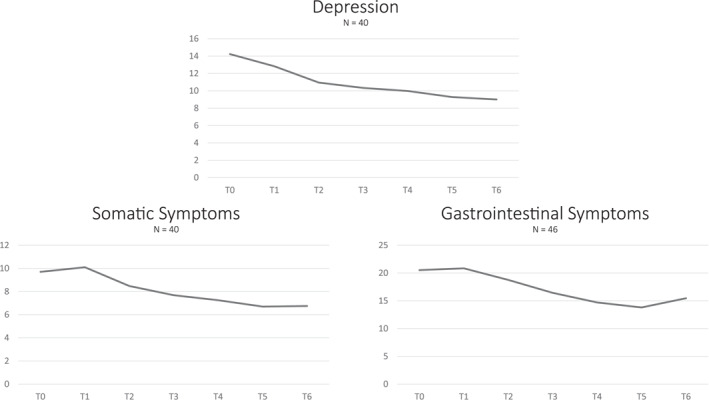
Course of symptoms during high‐caloric realimentation in anorexia nervosa (T0 = baseline, T1–T6 = week 1 to week 6 of treatment, respectively).

Adherence to the treatment regimen was generally good and increased significantly (lower values denote more adherence) from week three, while symptoms of depression, somatic complaints, and gastro‐intestinal complaints decreased (Table 2 and Figure 2). Compared to the baseline values, significant decreases started to show early in week 2 (depression and somatic symptoms) or week 3 (gastro‐intestinal complaints).

**TABLE 2:** Course of adherence, depression and somatic symptoms over high‐caloric refeeding in anorexia nervosa.

	T0 Mean (SD)	T1 Mean (SD)	T2 Mean (SD)	T3 Mean (SD)	T4 Mean (SD)	T5 Mean (SD)	T6 Mean (SD)	ANOVA F (df)	Comparisons versus T0				
										*t*	df	*p*	*d*
Adherence (*N* = 46)	1.28 (0.62)	1.26 (0.71)	1.20 (0.69)	1.09 (0.69)	1.02 (0.72)	0.96 (0.79)	0.91 (0.81)	6.50 (6; 270) *p* < 0.001	T1 versus T0	0.26	45	0.799	0.04
									T2 versus T0	1.00	45	0.323	0.15
									T3 versus T0	2.14	45	0.037	0.32
									T4 versus T0	2.73	45	0.009	0.40
									T5 versus T0	3.16	45	0.003	0.47
									T6 versus T0	3.25	45	0.002	0.48
Depression (*N* = 40)	14.23 (6.88)	12.83 (6.40)	10.95 (6.08)	10.33 (6.59)	9.98 (6.51)	.28 (6.25)	9.00 (6.62)	21.36 (6; 234) *p* < 0.001	T1 versus T0	2.00	39	0.053	0.32
									T2 versus T0	5.28	39	< 0.001	0.84
									T3 versus T0	5.48	39	< 0.001	0.87
									T4 versus T0	5.80	39	< 0.001	0.92
									T5 versus T0	6.45	39	< 0.001	1.02
									T6 versus T0	6.91	39	< 0.001	1.09
Somatic symptoms (*N* = 40)	9.70 (4.77)	10.10 (4.86)	8.48 (3.91)	7.68 (4.07)	7.25 (3.89)	6.70 (3.72)	6.75 (3.81)	15.44 (6; 234) *p* < 0.001	T1 versus T0	0.68	39	0.504	0.11
									T2 versus T0	2.25	39	0.030	0.36
									T3 versus T0	3.62	39	< 0.001	0.57
									T4 versus T0	4.50	39	< 0.001	0.71
									T5 versus T0	4.59	39	< 0.001	0.73
									T6 versus T0	5.66	39	< 0.001	0.90
Gastro‐intestinal symptoms (*N* = 46)	20.52 (14.96)	20.85 (16.55)	18.76 (15.77)	16.43 (14.18)	14.70 (13.87)	13.80 (15.03)	15.46 (15.55)	8.62270) *p* < 0.001	T1 versus T0	0.21	45	0.835	0.03
									T2 versus T0	1.12	45	0.268	0.17
									T3 versus T0	2.54	45	0.015	0.37
									T4 versus T0	3.77	45	< 0.001	0.56
									T5 versus T0	4.15	45	< 0.001	0.61
									T6 versus T0	2.97	45	0.005	0.44

*Note:* T0 = Baseline at beginning of treatment; T1–T6 = week 1‐week 6 of treatment (respectively).

Abbreviation: SD = standard deviation.

Age at admission (*r* = 0.342; *p* = 0.020) and rapid response (weight increase in the first 2 weeks of realimentation; *r* = 0.659; *p* < 0.001) emerged as significant correlates of BMI change over the initial 6‐week realimentation period. Table S2 shows the correlations for all variables included in this analysis. Phobic anxiety was the only significant correlate of the drive for thinness change score, indicating that more anxious patients showed more increase in drive for thinness (*r* = 0.332; *p* = 0.036). For the body dissatisfaction change score, only the SWLS score emerged as significant correlate. Patients with lower satisfaction with life at admission showed higher body dissatisfaction change scores (*r* = −0.346; *p* = 0.029).

## 4 Discussion

This is the first study to report on ED‐specific and psychological symptoms in weekly assessments over the course of a HCR programme in inpatients with AN. Over the first 6 weeks, body weight increased significantly each week during the realimentation, but drive for thinnesss scores did not change significantly and remained remarkably stable. Body dissatisfaction increased but with a delay relative to increased body weight. Depression and somatic symptoms decreased early in the realimentation programme. Out of a large number of potential correlates, only two (age at admission and rapid weight increase in the first 2 weeks) showed a significant correlation to body weight change during the initial phase of realimentation.

Drive for thinnesss did not change significantly over the 6‐weeks of realimentation in our study. This agrees with the study of Hemmingsen et al. (2022) who also reported no significant change in drive for thinnesss over 41 days of nutritional stabilisation. Similarly, the EDI total score showed only very little, and non‐significant change in the study of Riedlinger et al. (2022). Using a similar assessment, subscale scores of the Eating Disorder Examination Questionnaire measuring restraint and eating concerns decreased significantly during standard nutrition supported by nasogastric tube feeding (Kezelman et al. 2018).

Body dissatisfaction increased slightly but significantly, especially in the second half of the initial 6 weeks of our realimentation programme when body weight increases are increasingly perceptible to the patients. This is contrary to the finding of non‐significant increase of body dissatisfaction reported by Hemmingsen et al. (2022). A possible explanation is the substantially higher body dissatisfaction score in our patients at the beginning of treatment compared to the patients of Hemmingsen et al. (37.3 vs. 28.8).

Compared to baseline, depression decreased weekly and significantly from the second week of treatment. From week 4 mean depression scores were below 10, indicating a milder form of depression. Riedlinger et al. (2022)'s patients started treatment with lower PHQ‐depression scores which decreased from 10.76 to 8.21 over 9.5 weeks of treatment characterising a much less depressed sample than ours. Our findings are supported by several studies reporting a significant decrease of depression during realimentation treatment (Hemmingsen et al. 2022; Kezelman et al. 2018; Mattar et al. 2012; Salter et al. 2024). It is probable that the decrease of depression is not attributable to weight increase alone, as realimentation is generally embedded in a supportive inpatient treatment setting, contributing to improvement of emotional and cognitive symptoms.

Somatic and gastro‐intestinal symptoms also decreased significantly during the initial phase of treatment which included close medical monitoring with prescription of medication when necessary. This conforms to the results of other studies with similar assessments which also showed decreasing gastro‐intestinal symptoms over treatment for AN (Riedlinger et al. 2022; Waldholtz and Andersen 1990).

In accordance with other studies (Cuntz et al. 2022; Dalenbrook et al. 2022; Davies et al. 2017; Garber et al. 2012, 2013, 2021; Gjoertz et al., 2020; Golden et al. 2013; Koerner et al. 2020; Leclerc et al. 2013; Madden, Miskovic‐Wheatley, Clarke, et al. 2015a; Smith et al. 2016; Staab et al. 2022; Whitelaw et al. 2010) no refeeding syndrome was developed during the initial phase of rapid HCR. We attribute this to the close surveillance of clinical parameters and prophylactic substitution of phosphate and thiamine included in our realimentation programme as recommended by Garber et al. (2021), which proves once again that rapid weight gain can be safely achieved even in extremely underweight patients.

Varying durations of treatment, different clinical settings and weight gain make comparison of weight gain between studies difficult, and we recomputed reported weight gain as average kg per week (kg/wk) and average increase in BMI points per week (BMIpoints/wk). Over the course of the initial 6‐week realimentation programme our patients gained on the average 4.92 kg of body weight. The BMI increased by 1.79 kg/m^2^. For our study average weight increase was 0.82 kg/week or 0.30 BMI points/week. Two other studies reported a weight gain of 0.51 and 0.75 kg/wk (Hemmingsen et al. 2022; Dalenbrook et al. 2022; respectively), and three studies reported a weight gain of 0.19, 0.29, and 0.59 BMI points/wk (Riedlinger et al. 2022; Pettersson et al. 2016; Smith et al. 2016; respectively). However, there are important differences compared to our study. The patients of all these studies had a higher admission weight (BMI 13.1–15.9) than our patients. Two additional studies even included naso‐gastric tube feeding in addition to oral realimentation, making them not comparable to our study (Madden, Miskovic‐Wheatley, Clarke, et al. 2015a; Kezelman et al. 2018). Our observation period in this study was 6 weeks and the weight gain in this period was higher than the 0.51 kg/wk in the 6‐week period of the study of Hemmingsen et al. (2022). Concluding from these results, the first phase of our HCR programme resulted in very good improvement of symptoms without increasing psychological symptoms.

Out of a large number of potential correlates of BMI change over the initial realimentation period, only two variables emerged as significant. Higher age at admission was correlated to a higher increase of the BMI during treatment. This is contrary to the findings of Rienecke et al. (2025) who found higher weight gain in age groups less than 18 years and 18–25 years compared to age groups 26–39 years and 40 plus years. These authors concluded that there is a need for more specific treatments to increase weight gain in older adults. From our data we could conclude that HCR is a treatment suitable for older patients with AN without being unsuitable for younger patients. On the other hand, our sample showed a rather broad age range (13–62 years) and this could also account for the contrary finding. Age at admission showed no significant correlation to the number of previous inpatient or outpatient treatments, so we can exclude the possibility that higher age implied more chronicity. Regrettably, our data did not include age at onset or duration of illness, and we could not explore this possibility in more detail.

Weight increase in the first 2 weeks of realimentation was also correlated to a higher increase of the BMI during treatment. Although due to the intercorrelation of BMI values this finding has to be interpreted with caution, it fits well into the results of other studies which found early weight increase being a predictor for a better treatment outcome (Le Grange et al. 2014; Madden, Miskovic‐Wheatley, Wallis, et al. 2015b).

Strengths of the study include: (a) This is the first study to address psychological aspects over the course of rapid HCR with weekly assessments; (b) Only severe cases with very low body weight were included in the study; (c) This is a prospective study with an acceptable sample size. The same realimentation programme was offered to all participants, and we had not to rely on what was documented in the hospital charts by other medical carers; (d) There were few drop‐outs increasing representativeness of the sample.

Limitations include: (a) We report on the initial phase of one rapid realimentation programme in one clinic and it is not known if our findings extend beyond the first 6 weeks or apply to other HCR programs. On the other hand, treatment was offered to all patients without considering their type of health insurance and we have no evidence that our participants differed from other severe cases of AN treated elsewhere; (b) Due to ethical considerations we could not assign patients to a control group with treatment as usual, or a waiting list control group, so no comparison could be made between higher and lower caloric realimentation approaches; (c) We included predominantly assessments self‐rated by the participants. On the other hand, all self‐rated assessments were validated and published questionnaires meeting scientific standards.

## 5 Conclusion

For AN patients with extreme underweight HCR is a feasible and safe option. One important aim of such an intervention is to prepare the patient for an intensive psychotherapy. Under close medical monitoring the risk of developing a refeeding syndrome seems to be very low. One concern of a fast weight gain in AN is that increasing weight could trigger a rise in psychological symptoms like drive for thinnesss, body dissatisfaction or depression and gastrointestinal symptoms. Data from our and other studies do not support this concern to be valid. Although small increases in body dissatisfaction could be observed, these increases were within a manageable frame, and should not constitute an additional hurdle for efficient treatment. Further research should identify the special needs of demographic and diagnostic subgroups for this type of treatment.

## Funding

The authors have nothing to report.

## Ethics Statement

The study was reviewed and approved by the ethics committee of the University of Munich (no. 21‐0486; August 27, 2021).

## Conflicts of Interest

The authors declare no conflicts of interest.

## Supporting information


**Table S1:** Means and standard deviations of baseline scores.

**Table S2:** Pearson correlations of BMI difference T6–T0 with baseline variables.

## Data Availability

The data are not available due to data protection and legal restrictions.
