# Supplementary material for: High‐Caloric Realimentation and Mental and Physical Well‐Being in Patients With Extreme Anorexia Nervosa. A Prospective Study
Source: Eur Eat Disord Rev. 2025 Dec 25;34(3):835–44. doi: 10.1002/erv.70074 (PMC13048744; doi:10.1002/erv.70074)
Supplement: Supplementary file 1 — Table S1: Means and standard deviations of baseline scores. [file ERV-34-835-s002.docx]

Table S1: Means and standard deviations of baseline scores

| Age at admission | Mean | 27.57 |
| --- | --- | --- |
|  | Standard deviation | 10.89 |
|  | N | 46 |
| BMI change in the first two weeks of treatment | Mean | 0.70 |
|  | Standard deviation | 0.65 |
|  | N | 46 |
| EDI Drive for Thinness | Mean | 28.85 |
|  | Standard deviation | 10.21 |
|  | N | 46 |
| EDI Body Dissatisfaction | Mean | 37.33 |
|  | Standard deviation | 7.75 |
|  | N | 46 |
| Beck Depression Inventory-II score | Mean | 28.23 |
|  | Standard deviation | 12.91 |
|  | N | 43 |
| PHQ Depression | Mean | 14.23 |
|  | Standard deviation | 6.88 |
|  | N | 40 |
| PHQ Somatic Symptoms | Mean | 9.70 |
|  | Standard deviation | 4.77 |
|  | N | 40 |
| BSI Somatization | Mean | 0.93 |
|  | Standard deviation | 0.70 |
|  | N | 40 |
| BSI Obsessive-compulsive Symptoms | Mean | 1.48 |
|  | Standard deviation | 0.81 |
|  | N | 40 |
| BSI Interpersonal Sensitivity | Mean | 1.54 |
|  | Standard deviation | 0.97 |
|  | N | 40 |
| BSI Depression | Mean | 1.63 |
|  | Standard deviation | 0.92 |
|  | N | 40 |
| BSI Anxiety | Mean | 1.33 |
|  | Standard deviation | 0.84 |
|  | N | 40 |
| BSI Anger - Hostility | Mean | 0.82 |
|  | Standard deviation | 0.60 |
|  | N | 40 |
| BSI Phobic Anxiety | Mean | 0.57 |
|  | Standard deviation | 0.69 |
|  | N | 40 |
| BSI Paranoid Ideation | Mean | 0.88 |
|  | Standard deviation | 0.78 |
|  | N | 40 |
| BSI Psychoticism | Mean | 1.10 |
|  | Standard deviation | 0.88 |
|  | N | 40 |
| BSI General Symptomatic Index | Mean | 1.17 |
|  | Standard deviation | 0.67 |
|  | N | 40 |
| BSI Positive Symptom Total | Mean | 31.08 |
|  | Standard deviation | 10.56 |
|  | N | 40 |
| BSI Positive Symptom Distress Index | Mean | 1.88 |
|  | Standard deviation | 0.55 |
|  | N | 40 |
| Commitment to Exercise Scale total mean score | Mean | 2.51 |
|  | Standard deviation | 0.96 |
|  | N | 38 |
| Compulsive Exercise Test total mean score | Mean | 2.72 |
|  | Standard deviation | 1.29 |
|  | N | 38 |
| Brief Resilience Scale mean score | Mean | 2.81 |
|  | Standard deviation | 0.77 |
|  | N | 41 |
| Satisfaction With Life Scale sum score | Mean | 16.50 |
|  | Standard deviation | 7.66 |
|  | N | 40 |
| Gastro Questionnaire sum score | Mean | 20.52 |
|  | Standard deviation | 14.96 |
|  | N | 46 |
| Adherence to the treatment regimen | Mean | 1.28 |
|  | Standard deviation | 0.62 |
|  | N | 46 |
| Drive for exercise | Mean | 3.11 |
|  | Standard deviation | 2.12 |
|  | N | 46 |

EDI = Eating Disorder Inventory; PHQ = Patient Health Questionnaire; BSI = Brief Symptom Inventory
