# Supplementary material for: High‐Caloric Realimentation and Mental and Physical Well‐Being in Patients With Extreme Anorexia Nervosa. A Prospective Study
Source: Eur Eat Disord Rev. 2025 Dec 25;34(3):835–44. doi: 10.1002/erv.70074 (PMC13048744; doi:10.1002/erv.70074)
Supplement: Supplementary file 2 — Table S2: Pearson correlations of BMI difference T6–T0 with baseline variables. [file ERV-34-835-s001.docx]

Table S2: Pearson correlations of BMI difference T6 – T0 with baseline variables

| Age at admission | Pearson correlation | .342 |
| --- | --- | --- |
|  | Significance | .020 |
|  | N | 46 |
| BMI change in the first two weeks of treatment | Pearson correlation | .659 |
|  | Significance | <.001 |
|  | N | 46 |
| EDI Drive for Thinness | Pearson correlation | -.167 |
|  | Significance | .269 |
|  | N | 46 |
| EDI Body Dissatisfaction | Pearson correlation | -.077 |
|  | Significance | .613 |
|  | N | 46 |
| Beck Depression Inventory-II score | Pearson correlation | -.086 |
|  | Significance | .585 |
|  | N | 43 |
| PHQ Depression | Pearson correlation | -.028 |
|  | Significance | .863 |
|  | N | 40 |
| PHQ Somatic Symptoms | Pearson correlation | .127 |
|  | Significance | .433 |
|  | N | 40 |
| BSI Somatization | Pearson correlation | .264 |
|  | Significance | .100 |
|  | N | 40 |
| BSI Obsessive-compulsive Symptoms | Pearson correlation | .103 |
|  | Significance | .526 |
|  | N | 40 |
| BSI Interpersonal Sensitivity | Pearson correlation | -.202 |
|  | Significance | .212 |
|  | N | 40 |
| BSI Depression | Pearson correlation | .018 |
|  | Significance | .912 |
|  | N | 40 |
| BSI Anxiety | Pearson correlation | .115 |
|  | Significance | .478 |
|  | N | 40 |
| BSI Anger - Hostility | Pearson correlation | .142 |
|  | Significance | .382 |
|  | N | 40 |
| BSI Phobic Anxiety | Pearson correlation | .083 |
|  | Significance | .611 |
|  | N | 40 |
| BSI Paranoid Ideation | Pearson correlation | -.089 |
|  | Significance | .585 |
|  | N | 40 |
| BSI Psychoticism | Pearson correlation | -.031 |
|  | Significance | .850 |
|  | N | 40 |
| BSI General Symptomatic Index | Pearson correlation | .047 |
|  | Significance | .775 |
|  | N | 40 |
| BSI Positive Symptom Total | Pearson correlation | -.025 |
|  | Significance | .880 |
|  | N | 40 |
| BSI Positive Symptom Distress Index | Pearson correlation | .045 |
|  | Significance | .784 |
|  | N | 40 |
| Commitment to Exercise Scale total mean score | Pearson correlation | .178 |
|  | Significance | .284 |
|  | N | 38 |
| Compulsive Exercise Test total mean score | Pearson correlation | .076 |
|  | Significance | .650 |
|  | N | 38 |
| Brief Resilience Scale mean score | Pearson correlation | -.042 |
|  | Significance | .792 |
|  | N | 41 |
| Satisfaction With Life Scale sum score | Pearson correlation | .019 |
|  | Significance | .905 |
|  | N | 40 |
| Gastro Questionnaire sum score | Pearson correlation | .012 |
|  | Significance | .938 |
|  | N | 46 |
| Adherence to the treatment regimen | Pearson correlation | .009 |
|  | Significance | .954 |
|  | N | 46 |
| Drive for exercise | Pearson correlation | .236 |
|  | Significance | .115 |
|  | N | 46 |

EDI = Eating Disorder Inventory; PHQ = Patient Health Questionnaire;

BSI = Brief Symptom Inventory
